# Influence of Diabetes Mellitus on Perioperative Outcomes Following Surgical Stabilization of Rib Fractures: A National Health Insurance Research Database Analysis

**DOI:** 10.3390/medicina61081358

**Published:** 2025-07-26

**Authors:** Yang-Fan Liu, Te-Li Chen, Jian-Wei Guo, Shih-Ching Liu, Wen-Ching Wang

**Affiliations:** 1 Department of Traumatology, Hsinchu MacKay Memorial Hospital, Hsinchu 300044, Taiwan; 2 Department of Thoracic Surgery, Hsinchu MacKay Memorial Hospital, Hsinchu 300044, Taiwan; 3 Department of Life Science & Institute of Molecular and Cellular Biology, National Tsing Hua University, No. 690, Sec. 2, Guangfu Rd., East Dist., Hsinchu 300044, Taiwan; 4 International Intercollegiate Ph.D. Programme, National Tsing Hua University, Hsinchu 300044, Taiwan; 5 Department of Emergency, Hsinchu MacKay Memorial Hospital, Hsinchu 300044, Taiwan; 5688@mmh.org.tw (T.-L.C.);

**Keywords:** diabetes complications, surgical stabilization of rib fractures (SSRF), flail chest, postoperative outcomes, National Health Insurance Research Database (NHIRD)

## Abstract

*Background and Objectives:* Diabetes mellitus (DM) significantly impacts post-surgical recovery and fracture healing; however, few studies have specifically investigated the impact of DM on outcomes in patients undergoing surgical stabilization of rib fractures (SSRF). This study investigated the potential influence of DM on perioperative outcomes following SSRF, using data from Taiwan’s National Health Insurance Research Database (NHIRD). *Materials and Methods:* Data of 1603 patients with multiple rib fractures who underwent SSRF between 2001 and 2019 were retrospectively analyzed. Patients were categorized into three groups: no DM, DM without chronic complications, and DM with chronic complications. The associations between DM status and perioperative outcomes, including hospital length of stay (LOS), in-hospital mortality, readmission rates, and complications such as pneumonia, surgical site infection (SSI), acute myocardial infarction (AMI), and total hospital costs were determined using univariate and multivariable regression analyses. *Results:* The mean age of the 1603 patients was 52.0 years, and 71% were male. Patients with DM and chronic complications had higher risks of 14-day readmission (adjusted odds ratio [aOR] = 2.99; 95% confidence interval [CI]: 1.18–7.62), 15–30 day readmission (aOR = 3.28; 95% CI: 1.25–8.60), SSI (aOR = 2.90; 95% CI: 1.37–6.14), AMI (aOR = 3.44; 95% CI: 1.28–9.24), and acute respiratory distress syndrome (ARDS) (aOR = 1.96; 95% CI: 1.03–3.74). In conclusion, DM, particularly DM with chronic complications, significantly increases the risk of adverse short-term outcomes following SSRF. *Conclusions:* These findings emphasize the need for enhanced care for patients with DM to optimize the outcomes of SSRF.

## 1. Introduction

Diabetes mellitus (DM) is a chronic metabolic disorder and is associated with many complications, including an increased risk of fractures and impaired fracture healing [1]. Alterations in bone metabolism and microstructure resulting from DM significantly compromised the bone healing process [2,3,4]. A 2011 database analysis of 90.1 million patients in the United States (US) identified DM as a major risk factor for fracture nonunion, with an odds ratio (OR) of 1.40 [5]. Both insulin-dependent and non-insulin-dependent DM are associated with a significantly increased risk of impaired fracture healing, likely due to hyperglycemia-induced inflammation and fracture instability [6,7].

Hyperglycemia exacerbates oxidative stress through the production of reactive oxygen species (ROS) and inflammatory mediators such as interleukin (IL)-1β, IL-23, and tumor necrosis factor (TNF)-α, which disrupt the bone healing process [8]. It also promotes the formation of advanced glycation end products (AGEs), which bind to the receptor for advanced glycation end products (RAGEs) and further drive oxidative stress and inflammation, adversely affecting bone homeostasis [9,10,11]. Consequently, patients with DM experience significantly higher rates of delayed or nonunion fracture healing compared to controls, primarily due to disrupted balance between bone resorption and bone formation, resulting in reduced new bone formation at fracture sites [12,13].

Surgical stabilization of rib fractures (SSRF) has become a routine procedure at many high-volume trauma centers [14]. Although multiple rib fractures and flail chest have traditionally been managed with supportive care, an increasing number of these patients are now being treated with SSRF [15]. Recent studies have shown that SSRF improves postoperative outcomes for patients with flail chest, older patients, and certain individuals with multiple rib fractures [16,17]. However, studies indicated that DM is associated with longer hospital stay and increased complications following SSRF [18,19].

Recent guidelines emphasize the critical role of perioperative glycemic control in reducing postoperative complications among patients with diabetes. The American Diabetes Association (ADA) recommends maintaining blood glucose levels between 80 and 180 mg/dL during the perioperative period to minimize risks such as infection, delayed wound healing, and increased mortality [20]. Similarly, the Centre for Perioperative Care (CPOC) advocates for a multidisciplinary approach to perioperative care, highlighting the need for individualized glycemic targets and integration of diabetes management throughout the surgical pathway [21]. Advancements in diabetes management technologies, including continuous glucose monitoring (CGM) systems and insulin pump therapies, have also enabled tighter perioperative glycemic control, reducing glycemic variability and associated complications [22].

To date, few studies have specifically investigated the impact of DM, particularly with chronic complications, on postoperative outcomes in patients undergoing SSRF. To address this knowledge gap, this study aimed to examine the influence of DM, with and without chronic complications, on postoperative outcomes following SSRF, using data from the Taiwan National Health Insurance Research Database (NHIRD).

## 2. Methods

### 2.1. Data Source

Data from the Taiwan NHIRD were retrospectively reviewed [23]. The NHIRD was established by Taiwan’s National Health Insurance (NHI) program and is a robust population-based resource that includes data on over 23 million people, covering more than 99% of Taiwan’s population. This extensive database contains detailed healthcare records, such as patient demographics, outpatient visits, hospital admissions, prescription drug use, and disease diagnoses. For this study, diagnoses and medical classifications were identified using the International Classification of Diseases, Tenth Revision, Clinical Modification (ICD-9-CM/ICD-10-CM), and Procedure Coding System (ICD-9-PCS/ICD-10-PCS) codes.

### 2.2. Ethics Statement

This study received approval from MacKay Memorial Hospital, Taiwan (approval number: 23MMHIS340e). As this research used secondary data from a de-identified public insurance database, there was no direct involvement of patients, and the requirement for informed consent was waived.

### 2.3. Study Population

Patients aged ≥ 18 years old identified in the NHIRD who were admitted with multiple rib fractures or flail chest and underwent their first SSRF between 1 January 2001 and 31 December 2019 were included in the analysis. Patients with missing demographic data (age, sex, income, residential area), missing data on DM severity or comorbidities, or those with moderate to severe traumatic brain injury were excluded. Additionally, patients with prior SSRF for rib fractures before sustaining multiple rib fractures or flail chest were also excluded. The detailed ICD-9-CM/ICD-10-CM and ICD-9-PCS/ICD-10-PCS codes used in this study are provided in Supplementary Table S1.

### 2.4. Study Outcomes and Variables

Study outcomes included overall hospital length of stay (LOS), in-hospital mortality, readmission rates (within 14 days and 15–30 days), total hospital costs, and adverse outcomes such as pneumonia, surgical site infection (SSI), acute myocardial infarction (AMI), and respiratory failure/acute respiratory distress syndrome (ARDS).

Patients were classified into 3 groups: nondiabetic, DM without chronic complications, and DM with chronic complications. The chronic diabetic complications included diabetic nephropathy, diabetic retinopathy, diabetic cataract, diabetic neuropathy, and diabetic peripheral angiopathy. Baseline characteristics of the patients were extracted from the NHIRD, including age, sex, monthly income, fracture type, hospital region, and comorbidities such as hypertension, ischemic heart disease, congestive heart failure, anemia, chronic obstructive pulmonary disease (COPD), rheumatic disease, and any malignancy. The detailed ICD-9-CM/ICD-10-CM codes used for the study outcomes are provided in Supplementary Table S1.

### 2.5. Statistical Analysis

Descriptive statistics for the baseline characteristics were compared across the 3 patient groups (without DM, with DM but without chronic complications, and with DM and chronic complications). Continuous variables were presented as median (interquartile range, IQR) and analyzed using the Wilcoxon rank-sum test. Categorical variables were presented as count and percentage, and they were compared using the chi-square test or Fisher’s exact test, as appropriate. Univariate and multivariable logistic regression models were used to evaluate the associations between DM status and binary outcomes, such as readmission and adverse events. Additionally, univariate and multivariable linear regression analyses were conducted to examine the relations between DM, LOS (days), and total hospital costs (in thousands of New Taiwan Dollars, TWD), adjusting for significant covariates identified in the univariate analysis (excluding detailed comorbidities). All *p*-values were 2-sided, and a *p*-value of <0.05 was considered statistically significant. Statistical analyses were performed using SAS software, version 9.4 (SAS Institute Inc., Cary, NC, USA).

## 3. Results

### 3.1. Patient Selection

**Figure 1:**
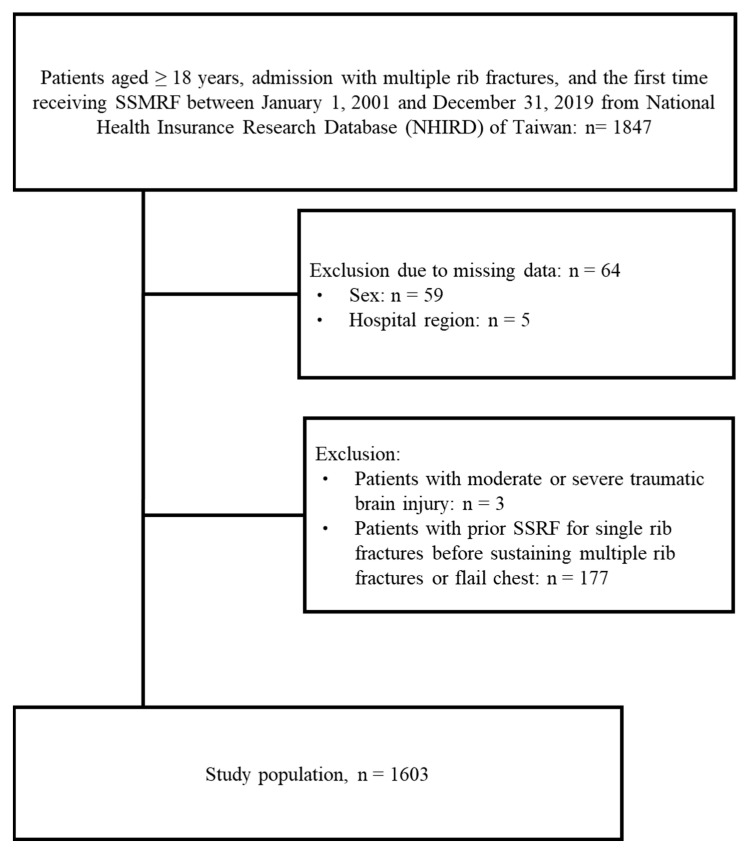
Flow chart of study population selection.

The flow diagram illustrating the selection of the study population is shown in Figure 1. A total of 1847 patients ≥ 18 years old diagnosed with multiple rib fractures and treated with SSRF between 1 January 2001 and 31 December 2019 were identified in the NHIRD. Patients with incomplete data regarding sex and hospital region (*n* = 64) were excluded. Additionally, those with moderate or severe traumatic brain injury (*n* = 3) and those with prior SSRF for rib fractures before sustaining multiple rib fractures or flail chest (*n* = 177) were also excluded. Ultimately, 1603 patients were included in the analysis (Figure 1).

### 3.2. Patient Characteristics

Patient demographic characteristics are summarized in Table 1. The median age of all patients was 52.0 years, and 71% were male. Approximately 70% of the patients had a low monthly income (income < TWD 20,000), and 9% of the patients had more than three comorbidities. Compared to patients without DM and those with DM without complications, the group of patients with DM with chronic complications was the oldest (median age: 62.5 years vs. 51.0–58.0 years) and had the highest percentage of patients with more than three comorbidities (25.6% vs. 6.8–15.3%). Of the comorbidities, hypertension was the most common in patients with DM and chronic complications (75.6% vs. 23.1–56.9%). Additionally, patients with DM without chronic complications had the highest percentage of monthly income < TWD 20,000 (75.1% vs. 67.4–70.7%) (Table 1).

### 3.3. Associations Between DM and Perioperative Outcomes

The perioperative outcomes and complications of the three groups are summarized in Table 2. Compared to patients without DM and those with DM but without complications, patients with DM and chronic complications had significantly higher rates of 14 day (9.5%) and 15–30 day readmission (9.5%), SSI (11%), AMI (8.5%), and ARDS (18%). Patients with DM without chronic complications had the highest total hospital costs and higher percentages of in-hospital mortality (10%) and pneumonia (18%) (Table 2).

**Figure 2:**
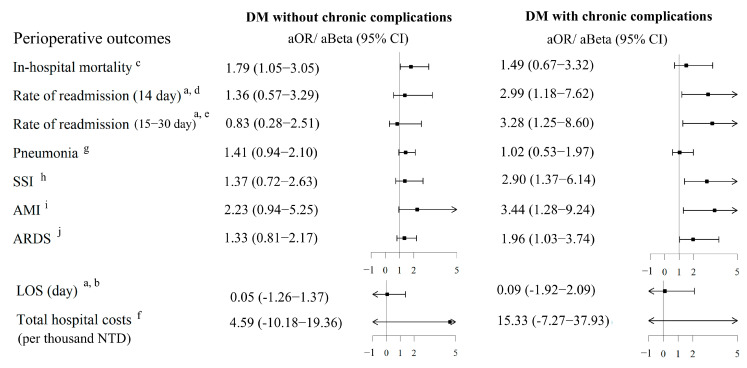
Forest plot for adjusted associations between DM with and without chronic complications, and perioperative outcomes. The arrow symbol indicates that the 95% confidence interval extends beyond the lower or upper limit of the axis. SSI, surgical site infection; AMI, acute myocardial infarction; ARDS, acute respiratory distress syndrome; LOS, length of stay; TWD, New Taiwan Dollar. ^a^ Excluded patients who died in the hospital. ^b^ Adjusted for related variables of *p* < 0.05 in univariate analysis (except comorbidities), including age (continuous), sex, flail chest, monthly income, and number of comorbidities. ^c^ Adjusted for related variables of *p* < 0.05 in univariate analysis (except comorbidities), including age (continuous) and sex. ^d^ Adjusted for related variables of *p* < 0.05 in univariate analysis (except comorbidities), including hospital region and number of comorbidities. ^e^ Adjusted for related variables of *p* < 0.05 in univariate analysis (except comorbidities), including age (continuous) and number of comorbidities. ^f^ Adjusted for related variables of *p* < 0.05 in univariate analysis (except comorbidities), including age (continuous), flail chest, and number of comorbidities. ^g^ Adjusted for related variables of *p* < 0.05 in univariate analysis (except comorbidities), including age (continuous) and monthly income. ^h^ Adjusted for related variables of *p* < 0.05 in univariate analysis (except comorbidities), including monthly income. ^i^ Adjusted for related variables of *p* < 0.05 in univariate analysis (except comorbidities), including age (continuous) and number of comorbidities. ^j^ Adjusted for related variables of *p* < 0.05 in univariate analysis (except comorbidities), including age (continuous), monthly income, and number of comorbidities.

After adjusting for other covariates in multivariable analyses, the estimated associations between DM and perioperative outcomes and complications are shown in Figure 2. We observed that patients with DM without chronic complications had a significantly higher risk for in-hospital mortality (adjusted odds ratio [aOR] = 1.79, 95% CI: 1.05–3.05, *p* = 0.032) than those without DM. Patients with DM with chronic complications had a significantly higher risk for 14-day readmission (aOR = 2.99, 95% CI: 1.18–7.62, *p* = 0.022), 15–30-day readmission (aOR = 3.28, 95% CI: 1.25–8.60, *p* = 0.016), SSI (aOR = 2.90, 95% CI: 1.37–6.14, *p* = 0.006), AMI (aOR = 3.44, 95% CI: 1.28–9.24, *p* = 0.015), and ARDS (aOR = 1.96, 95% CI: 1.03–3.74, *p* = 0.041) than those without DM (Figure 2). Detail results of univariate and multivariable analyses are also summarized in Supplementary Table S2.

## 4. Discussions

Our study demonstrates that DM significantly influences perioperative outcomes in patients undergoing SSRF. The results indicate that compared to patients without DM, those with DM without complications have a 1.8 times higher risk of in-hospital mortality. Importantly, compared to patients without DM, those with DM and complications have about 3 times higher risks of 14 day and 15–30 day readmission, as well as 2.9 times higher risk of SSI, 3.4 times higher risk of AMI, and 2 times higher risk of ARDS. These findings underscore the necessity for heightened vigilance and targeted interventions in patients with DM and chronic complications to mitigate these risks and improve their overall surgical outcomes.

Limited studies have explored the factors influencing the outcomes of SSRF. A previous study has documented that the severity of the initial trauma remains a significant risk factor for delayed extubation and increased complication rates, even after SSRF [24]. Additionally, the timing of the procedure remains a topic of debate [25]. Among the potential risk factors, our previous study underscored the association between a higher body mass index (BMI) and poorer outcomes following SSRF [26]. Nevertheless, continuous investigations into postoperative outcome factors are still needed to improve preoperative risk stratification and optimize perioperative care.

Diabetes mellitus has become a global health issue secondary to the obesity epidemic, and it has been long known that DM is associated with complications of most physiological systems of the body including cardiovascular, renal, and nerve diseases [27]. DM is rarely reversible, and while there are many glucose-lowering medications, even with tight glucose control, a person still remains at risk of diabetic complications [27]. Although the pathophysiological mechanisms of many diabetic complications have yet to be completely understood, recent studies are indicating that oxidative stress plays an important role [3,4,27].

Impaired fracture healing and decreased “bone health” are now recognized as complications of DM. Recent studies have shown that long-term increased glucose levels alter the bone microvasculature, resulting in decreased bone mass and impaired healing [28,29]. Two recent systematic reviews and meta-analyses concluded that DM markedly impairs fracture healing and increases the risks of infection, malunion, nonunion, and the need for reoperation [30,31]. This motivated our present analysis, which aims to further investigate whether diabetes mellitus has an impact on the outcomes following SSRF for multiple rib fractures.

Our study extends the findings of prior research showing that DM is associated with poor fracture healing and increased complications following various surgical procedures [2,18,32] by demonstrating that DM, especially DM with complications, is independently associated with worse postoperative outcomes in patients with rib fractures who undergo SSRF. These results are consistent with previous studies reporting longer hospital stays and higher complication rates in patients with DM following fracture and fracture repair [2,18,32,33].

Especially notable about our findings is that patients with DM with established chronic complications had a significantly higher incidence of SSI, AMI, and ARDS. Another study has shown that DM with complications is associated with worse outcomes than DM without complications. For example, compared to patients with uncomplicated DM, those with DM and complications undergoing carotid endarterectomy had significantly higher risks of AMI, stroke, infection, and death [34]. The increased risks of SSI, AMI, and ARDS in patients with DM and complications are likely, in part, due to the overproduction of reactive oxygen species (ROS) and the accumulation of advanced glycation end products (AGEs) as a result of persistently elevated glucose levels [3,4]. Excessive ROS and AGEs have been shown to activate inflammatory cascades and damage the micro- and macrovasculature, thus predisposing patients to complications such as SSI and AMI [3,4].

Specific to our findings of increased risk of SSI, a review by Wang et al. [35] described how ROS and AGEs delay wound healing by impairing immune function and causing endothelial cell dysfunction, both of which increase the risk of infections and slow tissue repair at surgical sites. Furthermore, Moris et al. [36] reviewed the relationship between ROS and cardiovascular disease. An increase in ROS results in redox imbalance, oxidative stress, and cell injury, which lead to damage of the endothelial lining of blood vessels, which promotes inflammation and atherosclerosis [36]. In addition, AGEs stiffen arteries and destabilize plaques, which increases the risk of plaque rupture, subsequently leading to an AMI [36]. Similarly, in the lungs, ROS cause tissue damage and increase alveolar permeability, and the accumulation of AGEs promotes inflammation, and ultimately, these changes contribute to ARDS due to impaired gas exchange and fluid accumulation [37].

In our study, we grouped patients with DM into those without complications and those with chronic complications, and the results demonstrated that the presence of chronic complications was a significant risk factor for readmission, whereas patients with DM without complications were not at increased risk of readmission. Few studies have examined readmission rates, and this finding underscores the impact of disease progression on outcomes. In a study that did examine readmission rates, Liu et al. [38] reported that DM was associated with significantly greater readmission and reoperation rates, as well as increased mortality in patients undergoing open reduction and internal fixation (ORIF) of ankle fractures. The finding of higher readmission rates in patients with DM is not unexpected as the presence of hyperglycemia, oxidative stress, and inflammation associated with DM compromised the healing process and thus could ultimately result in higher readmission rates [6,31].

It is worth noting that our analysis revealed that SSRF patients with DM but without chronic complications exhibited a significantly higher risk of in-hospital mortality compared to non-DM patients. Conversely, while SSRF patients with DM and chronic complications also demonstrated an elevated risk of in-hospital mortality relative to nondiabetic patients, this difference did not reach statistical significance. There are several explanations for the inconsistency between these results and our hypothesis. First, the limited sample size of SSRF patients with DM and chronic complications may lead to reduced statistical power to detect significance, with narrower confidence intervals. Secondly, undiagnosed chronic illness may happen and exacerbate the severity of acute events in the group of SSRF patients with DM but without chronic complications, especially among patients lacking regular medical follow-up, further increasing in-hospital mortality rates [39]. Third, surgeons may choose more conservative or lower-risk surgical strategies for SSRF patients with diabetes mellitus and chronic complications, given their elevated baseline risks, potentially leading to lower postoperative mortality [40]. Further, SSRF patients with DM and chronic complications often receive more stringent medical monitoring during hospitalization, are typically managed by multiple specialists, and undergo more detailed preoperative risk assessments and postoperative monitoring plans, ensuring better outcomes with enhanced comprehensiveness of care [41,42,43,44].

Taken together, our study reveals that DM patients with chronic complications experience significantly higher rates of postoperative complications following SSRF compared to non-DM patients. Although it also shows that patients with DM but without chronic complications paradoxically have a higher risk of in-hospital mortality, our findings highlight the need for targeted perioperative strategies to improve outcomes in diabetic patients undergoing SSRF. Preoperative optimization of glycemic control, thorough assessment for diabetes-related complications, and early surgical intervention are critical components [45,46]. Implementing these strategies may mitigate the increased risks of complications such as surgical site infections, acute myocardial infarction, and acute respiratory distress syndrome observed in this patient population. By integrating these recommendations into clinical practice, healthcare providers can enhance the perioperative care of diabetic patients undergoing SSRF, potentially improving surgical outcomes and reducing morbidity.

With respect to SSRF, future studies should focus on prospective, controlled trials to further elucidate the impact of glycemic control and specific antidiabetic treatments on postoperative outcomes in SSRF patients. Additionally, interventions aimed at reducing perioperative risks in DM patients, such as improved preoperative optimization and postoperative monitoring, should be explored. Research into the mechanisms of fracture healing in the context of DM may also provide insight into novel therapeutic targets to enhance recovery and reduce complications in this high-risk population.

### Strengths and Limitations

One strength of our study is the use of a large, national database from Taiwan, the NHIRD, which provides comprehensive patient data and allows for an analysis over an extended period. Additionally, we carefully considered patient demographics and comorbidities, providing a more accurate assessment of the impact of DM on perioperative outcomes. However, our study has some limitations. As a retrospective analysis, it is subject to potential biases related to data collection and patient selection, including the exclusion of 244 patients based on predefined criteria. Future research with broader access to data should incorporate sensitivity analyses to assess the impact of such exclusions and enhance the generalizability of findings. Moreover, the NHIRD lacks several clinical details such as laboratory data (e.g., serum glucose and glycated hemoglobin [HbA1c] levels), intraoperative parameters (e.g., operative time, estimated blood loss, anesthesia type, and adjunct perioperative interventions), and postoperative care variables (e.g., ICU management, and analgesic use). The absence of these data limits our ability to adjust for disease severity and perioperative risk more precisely and may introduce residual confounding. Future studies linking claims data with those lacking data are warranted to better clarify their association. While we did not include specific covariates such as the Injury Severity Score (ISS), hemothorax, or pneumothorax due to data limitations in the NHIRD, we included flail chest as a proxy variable for thoracic injury severity. Additionally, our models were also adjusted for important demographic and clinical variables, including age, sex, comorbidity burden, income, and hospital region, to minimize potential confounding. Nevertheless, the absence of detailed trauma-related clinical information remains a limitation. Future studies should incorporate these potential confounders into the analysis to address this issue more comprehensively. Lastly, this study is limited to patients in Taiwan, and the results may not be fully applicable to other healthcare settings or populations.

## 5. Conclusions

This study highlights the significant impact of DM, particularly with chronic complications, on perioperative outcomes in patients undergoing SSRF. Patients combined with DM and chronic complications are at increased risk of postoperative complications such as SSI, AMI, and ARDS. These findings emphasize the need for proper perioperative management strategies and vigilant postoperative care in patients with DM, especially those with chronic complications, to improve surgical outcomes. Furthermore, this information could help inform surgeons and patients for better risk stratification and personalized treatment planning. Further research is needed to explore the role of glycemic control and other intraoperative factors to better understand and mitigate these risks.

## Figures and Tables

**Table 1:** Characteristics of patients who underwent SSRF by DM and chronic complications.

	Total (*n* = 1603)	Without DM	DM Without Chronic Complications	DM with Chronic Complications	*p*
		(*n* = 1312)	(*n* = 209)	(*n* = 82)	
**Demography**					
Age, years	52.0 (44.0–61.0)	51.0 (42.0–59.0)	58.0 (51.0–65.0)	62.5 (55.0–69.0)	**<0.001**
18–39	254 (15.8)	243 (18.5)	8 (3.8)	3 (3.7)	**<0.001**
40–59	876 (54.6)	742 (56.6)	106 (50.7)	28 (34.1)	
≥60	473 (29.5)	327 (24.9)	95 (45.5)	51 (62.2)	
Sex					0.815
Male	1131 (70.6)	926 (70.6)	145 (69.4)	60 (73.2)	
Female	472 (29.4)	386 (29.4)	64 (30.6)	22 (26.8)	
Monthly income, TWD ^a^					**0.004**
>25,000	349 (21.8)	309 (23.6)	27 (12.9)	13 (15.9)	
20,000–25,000	155 (9.7)	119 (9.1)	25 (12.0)	11 (13.4)	
<20,000	1099 (68.6)	884 (67.4)	157 (75.1)	58 (70.7)	
Flail chest	48 (3.0)	34 (2.6)	10 (4.8)	4 (4.9)	0.133
Hospital region					0.490
Urban	834 (52.0)	680 (51.8)	115 (55.0)	39 (47.6)	
Non-urban	769 (48.0)	632 (48.2)	94 (45.0)	43 (52.4)	
**Number of Comorbidities**					**<0.001**
0	898 (56.0)	834 (63.6)	55 (26.3)	9 (11.0)	
1	380 (23.7)	282 (21.5)	73 (34.9)	25 (30.5)	
2	183 (11.4)	107 (8.2)	49 (23.4)	27 (32.9)	
3+	142 (8.9)	89 (6.8)	32 (15.3)	21 (25.6)	
**Comorbidities**					
Hypertension	484 (30.2)	303 (23.1)	119 (56.9)	62 (75.6)	**<0.001**
Ischemic heart disease	231 (14.4)	140 (10.7)	56 (26.8)	35 (42.7)	**<0.001**
Congestive heart failure	75 (4.7)	46 (3.5)	19 (9.1)	10 (12.2)	**<0.001**
Anemia	83 (5.2)	60 (4.6)	16 (7.7)	7 (8.5)	0.065
COPD	212 (13.2)	151 (11.5)	43 (20.6)	18 (22.0)	**<0.001**
Rheumatic disease	60 (3.7)	42 (3.2)	12 (5.7)	6 (7.3)	**0.043**
Any malignancy	73 (4.6)	48 (3.7)	16 (7.7)	9 (11.0)	**0.001**

DM, diabetes mellitus; COPD, chronic obstructive pulmonary disease; TWD, New Taiwan Dollar. Continuous variables are presented as median (25–75th percentile) and compared using the Wilcoxon rank-sum test. Categorical variables are presented as number (%) and compared using the chi-square test or Fisher’s exact test, as appropriate. *p* < 0.05 shown in bold. ^a^ Monthly income was categorized in USD as follows: >USD 806.5, USD 645.2–806.5, and <USD 645.2.

**Table 2:** Perioperative outcomes and complications in patients undergoing SSRF by DM and chronic complications.

Perioperative Outcomes	Total (*n* = 1603)	Without DM	DM Without Chronic Complications	DM with Chronic Complications	*p*
		(*n* = 1312)	(*n* = 209)	(*n* = 82)	
LOS, day ^a^	8.0 (5.0–12.0)	8.0 (5.0–11.0)	9.0 (5.0–13.0)	8.0 (5.0–13.0)	0.088
In-hospital mortality	92 (5.7)	63 (4.8)	21 (10.0)	8 (9.8)	**0.003**
Rate of readmission (14 day) ^a^	42 (2.8)	28 (2.2)	7 (3.7)	7 (9.5)	**0.001**
Rate of readmission (15–30 day) ^a^	35 (2.3)	24 (1.9)	4 (2.1)	7 (9.5)	**0.002**
Total hospital costs, per thousand TWD	40.9 (28.8–86.6)	39.3 (28.6–81.3)	47.2 (30.6–108.7)	46.8 (32.0–130.4)	**0.001**
Pneumonia	204 (12.7)	154 (11.7)	38 (18.2)	12 (14.6)	**0.030**
SSI	74 (4.6)	53 (4.0)	12 (5.7)	9 (11.0)	**0.010**
AMI	34 (2.1)	18 (1.4)	9 (4.3)	7 (8.5)	**<0.001**
ARDS	136 (8.5)	96 (7.3)	25 (12.0)	15 (18.3)	**<0.001**

DM, diabetes mellitus; LOS, length of stay; TWD, New Taiwan Dollar; SSI, surgical site infection; AMI, acute myocardial infarction; ARDS, acute respiratory distress syndrome. Continuous variables are presented as median (25–75th percentile) and compared using the Wilcoxon rank-sum test. Categorical variables are presented as number (%) and compared using the chi-square test or Fisher’s exact test, as appropriate. *p* < 0.05 shown in bold. ^a^ Excluded patients who died in the hospital.

## Data Availability

The datasets used and/or analyzed in this study are available from the corresponding author on reasonable request.

## Supplementary Materials

The following supporting information can be downloaded at: https://www.mdpi.com/article/10.3390/medicina61081358/s1, Table S1: ICD codes used to define diseases and procedures; Table S2: Risks of complications in patients with DM with and without chronic complications compared to patients without DM estimated by univariate and multivariable analyses.
