# Supplementary material for: Influence of Diabetes Mellitus on Perioperative Outcomes Following Surgical Stabilization of Rib Fractures: A National Health Insurance Research Database Analysis
Source: Medicina (Kaunas). 2025 Jul 26;61(8):1358. doi: 10.3390/medicina61081358 (PMC12387593; doi:10.3390/medicina61081358)
Supplement: Supplementary file 1 [file medicina-61-01358-s001.zip › medicina-3657439-supplementary.pdf]

Supplementary Table S1: ICD codes used to define diseases and procedures

	ICD-9-CM / ICD-9-PCS	ICD-10-CM / ICD-10-PCS
Multiple rib fracture	807.02-807.09, 807.12-807.19	S22.4
Flail chest	807.4	S22.5
Diabetes mellitus without chronic complication	250.0-250.3, 250.8, 250.9	E10.0, E10.l, E10.6, E10.8, E10.9, E11.0, E11.1, E11.6, E11.8, E11.9, E12.0, E12.1, E12.6, E12.8, E12.9, E13.0, E13.1, E13.6, E13.8, E13.9, E14.0, E14.1, E14.6, E14.8, E14.9
Diabetes mellitus with chronic complication	250.4-250.7	E10.2-E10.5, E10.7, E11.2-E11.5, E11.7, E12.2-E12.5, E12.7, E13.2-E13.5, E13.7, E14.2-E14.5, E14.7
SSMRF	ICD-9-PCS: 84.94, 78.51, 79.19, 79.39	ICD-10-PCS: 0PH000Z, 0PH004Z, 0PH030Z, 0PH034Z, 0PH040Z, 0PH0 44Z, 0PH104Z, 0PH134Z, 0PH144Z, 0PH204Z, 0PH234Z, 0PH244Z, 0PH504Z, 0PH534Z, 0PH544Z, 0PH604Z, 0PH634Z, 0PH644Z, 0PH704Z, 0PH734Z, 0PH744Z, 0PH804Z, 0PH834Z, 0PH844Z, 0PH904Z, 0PH934Z, 0PH944Z, 0PHB04Z, 0PHB34Z, 0PHB44Z, 0PS000Z, 0PS030Z, 0PS040Z, 0PS034Z, 0PS044Z, 0PS134Z, 0PS144Z, 0PS234Z, 0PS244Z, 0PS534Z, 0PS544Z, 0PS634Z, 0PS644Z, 0PS734Z, 0PS744Z, 0PS834Z, 0PS844Z, 0PS934Z, 0PS944Z, 0PSB34Z, 0PSB44Z, 0QS234Z, 0QS244Z, 0QS334Z, 0QS344Z, 0QS434Z, 0QS444Z, 0QS534Z, 0QS544Z,

		0QSD34Z, 0QSD44Z, 0QSF34Z, 0QSF44Z, 0PS104Z, 0PS204Z
Moderate or severe traumatic brain injury	854.03, 854.04, 854.05	R40.242-R40.243
Pneumonia	073.0, 115.15, 115.95, 480, 481, 482, 483, 484.7, 484.8, 485, 486, 514, 517	J12–J18
SSI	998.5	T81.4
AMI	410	I21
ARDS	518.5, 518.81-518.84	J80, J81.0, J95.2-J95.8, J96.00, J96.90
Hypertension	401-405	I10, I11–I13, I15
Ischemic heart disease	410–414	I25
Congestive heart failure	398.90, 402.01, 402.11, 402.91, 404.01, 404.11, 404.91, 404.03 404.13, 404.93, 414.8, 425.2, 425.4, 425.5, 425.7, 425.8, 425.9, 428.0, 428.1, 428.9, 779.8	I09.9, I11.0, I13.0, I13.2, I25.5, I42.0, I42.5-I42.9, I43, I50, P29.0
Anemia	284.0, 284.8, 285.21, 285.22, 285.29, 285.0, 285.8, 285.9	D60, D61, D63, D64
COPD	490, 491.0, 491.1, 491.8, 491.9, 492.0, 492.8, 491.20, 491.21, 493.20, 493.21, 493.22, 496	J40-J44
Rheumatic disease	446.5, 710.0-710.4, 714.0-714.2, 714.8, 725	M05, M06, M31.5, M32-M34, M35.1, M35.3, M36.0

Any malignancy	140-172, 174-195, 200-208, 238.6, 196-199	C00-C26, C30-C34, C37-C41, C43, C45-C58, C60-C85, C88, C90-C97

SSMRF, surgical stabilization of multiple rib fractures; SSI, surgical site infection; AMI, acute myocardial infarction; ARDS, acute respiratory distress syndrome; COPD, chronic obstructive pulmonary disease.

Supplementary Table S2. Risks of complications in patients with DM with and without chronic complications compared to patients without DM.

	DM without chronic complications Vs. Non-DM		DM with chronic complications Vs. Non-DM		DM without chronic complications Vs. Non-DM		DM with chronic complications Vs. Non-DM	
**Perioperative outcomes**	OR/Beta [a] (95% CI)	*p*	OR/Beta [a] (95% CI)	*p*	aOR/aBeta [a] (95% CI)	*p*	aOR/aBeta [a] (95% CI)	*p*
LOS, day [b, c]	0.74 (-0.53-2.01)	0.253	0.94 (-1.00-2.89)	0.341	0.05 (-1.26-1.37)	0.935	0.09 (-1.92-2.09)	0.933
In-hospital mortality [d]	**2.21 (1.32-3.71)**	**0.003**	2.14 (0.99-4.64)	0.053	**1.79 (1.05-3.05)**	**0.032**	1.49 (0.67-3.32)	0.325
Rate of readmission (14 day) [b, e]	1.69 (0.73-3.92)	0.224	**4.56 (1.92-10.81)**	**<0.001**	1.36 (0.57-3.29)	0.491	**2.99 (1.18-7.62)**	**0.022**
Rate of readmission (15-30 day) [b, f]	1.11 (0.38-3.23)	0.849	**5.33 (2.22-12.82)**	**<0.001**	0.83 (0.28-2.51)	0.747	**3.28 (1.25-8.60)**	**0.016**
Total hospital costs, per thousand NTD [g]	13.81 (-0.55-28.17)	0.059	**29.59 (7.65-51.53)**	**0.008**	4.59 (-10.18-19.36)	0.543	15.33 (-7.27-37.93)	0.184
Pneumonia [h]	**1.67 (1.13-2.47)**	**0.010**	1.29 (0.68-2.43)	0.433	1.41 (0.94-2.10)	0.097	1.02 (0.53-1.97)	0.944
SSI [i]	1.45 (0.76-2.76)	0.261	**2.93 (1.39-6.17)**	**0.005**	1.37 (0.72-2.63)	0.337	**2.90 (1.37-6.14)**	**0.006**
AMI [j]	**3.24 (1.43-7.30)**	**0.005**	**6.71 (2.72-16.56)**	**<0.001**	2.23 (0.94-5.25)	0.068	**3.44 (1.28-9.24)**	**0.015**
ARDS [k]	**1.72 (1.08-2.74)**	**0.023**	**2.84 (1.56-5.15)**	**<0.001**	1.33 (0.81-2.17)	0.263	**1.96 (1.03-3.74)**	**0.041**

OR, odds ratio; aOR, adjusted odds ratio; CI, confidence interval; LOS, length of stay; SSI, surgical site infection; AMI, acute myocardial infarction; ARDS, acute respiratory distress syndrome.

$p < 0.05$ were showed in bold.

[a] Beta was used to LOS (day) and total hospital cost (per thousand NTD).

[b] Excluded patients who died in the hospital.

[c] Adjusted for related variables of p-value <0.05 in univariate analysis (except the comorbidities), including age (continuous), sex, flail chest, monthly income, and number of comorbidities.

[d] Adjusted for related variables of p-value <0.05 in univariate analysis (except the comorbidities), including age (continuous) and sex.

[e] Adjusted for related variables of p-value <0.05 in univariate analysis (except the comorbidities), including hospital region and number of comorbidities.
[f] Adjusted for related variables of p-value <0.05 in univariate analysis (except the comorbidities), including age (continuous) and number of comorbidities.
[g] Adjusted for related variables of p-value <0.05 in univariate analysis (except the comorbidities), including age (continuous), flail chest, and number of comorbidities.
[h] Adjusted for related variables of p-value <0.05 in univariate analysis (except the comorbidities), including age (continuous) and monthly income.
[i] Adjusted for related variables of p-value <0.05 in univariate analysis (except the comorbidities), including monthly income.
[j] Adjusted for related variables of p-value <0.05 in univariate analysis (except the comorbidities), including age (continuous) and number of comorbidities.
[k] Adjusted for related variables of p-value <0.05 in univariate analysis (except the comorbidities), including age (continuous), monthly income, and number of comorbidities.
